# Adhesion to stromal cells mediates imatinib resistance in chronic myeloid leukemia through ERK and BMP signaling pathways

**DOI:** 10.1038/s41598-017-10373-3

**Published:** 2017-08-25

**Authors:** Atul Kumar, Jina Bhattacharyya, Bithiah Grace Jaganathan

**Affiliations:** 1 Department of Biosciences and Bioengineering, Indian Institute of Technology Guwahati, Assam, 781039 India; 2 Department of Hematology, Gauhati Medical College, Assam, India

## Abstract

Chronic myeloid leukemia (CML) is characterized by abnormal proliferation of myeloid cells which when untreated leads to bone marrow failure. Imatinib mesylate (IM) is the first line of therapy for treatment of CML and results in remission in most cases. However, a significant percentage of patients develop chemoresistance to IM, which might be due to the presence of chemoresistant cells in the bone marrow. In the current study, we explored the role of cell-cell interaction of CML cells with the bone marrow stromal cells in the development of chemoresistance in CML. We found that the stromal cells offered long-term chemoprotection to the CML cells from the apoptotic effect of IM. These stroma interacting CML cells were maintained in a non-proliferative stage and had increased ERK1/2 and SMAD1/8 phosphorylation levels. Prolonged interaction of CML cells with the stromal cells in the presence of IM resulted in the acquisition of stroma-free chemoresistance to IM treatment. However, inhibition of actin cytoskeleton, ERK1/2 and SMAD signaling abrogated the chemoresistance acquisition and sensitized the chemoresistant CML cells to IM induced apoptosis.

## Introduction

Chronic myeloid leukemia (CML) is a myelo-proliferative disorder resulting in abnormally high number of myeloid cells in the bone marrow (BM)^1^. CML is initiated by reciprocal translocation t(9;22)(q34;q11) between chromosome 9 and chromosome 22 in hematopoietic stem cells (HSC). The resultant BCR-ABL protein lacks auto-inhibitory regulations and is a constitutively active tyrosine kinase^2^. BCR-ABL tyrosine kinase activity is essential for tumorigenesis^3^ and regulates RAS-MAPK-ERK^4, 5^, JNK-MAPK^6^, PI3K^7^, and STAT5^8, 9 ^signaling pathways in CML cells. These signaling pathways provide proliferative advantage to CML cells and also regulate anti-apoptotic genes. Tyrosine kinase inhibitors (TKIs) such as Imatinib mesylate (IM) which inhibit BCR-ABL kinase activity are used as frontline drug for chronic phase CML (CP-CML).

However, after few years of remission, a significant percentage of patients develop chemoresistance against IM. This percentage is higher in case of discontinuation of IM intake after CML remission. Mutations in the catalytic domain of BCR-ABL, which affects the binding ability of IM were initially reported to be the major cause of CML chemoresistance. However, increasing evidence indicate that BM microenvironment cells play a vital role in CML chemoprotection against TKIs.

BM stromal cells were reported by several groups to provide chemoprotection to CML cells via secreted factors. Stromal cell conditioned media (CM) and inflammatory cytokines such as IL-6, IL-8 secreted by the stromal cells protected CML cells from inhibitory effect of IM^10, 11^. Stromal cells secreted CM was reported to induce STAT3 activation and increased levels of anti-apoptotic regulators in CML cells^12^. Increased ERK activity in CML cells was reported upon exposure to FGF2, a stromal cell secreted cytokine^13^. However, direct cell-cell interaction seems to play a more important role in leukemia chemoprotection. CML cells adherent to the stromal compartments might evade chemotherapy resulting in minimal residual disease (MRD) and later relapse. CML –stromal cell interaction via VLA-4-VCAM-1 resulted in PlGF secretion from stromal cells which in turn supported CML cells in mouse BM^14^. N-Cadherin dependent cell-cell interaction was also implicated in stroma mediated chemoprotection in CML^15^.

Oncogene independent signaling pathways involved in stroma mediated chemoprotection of CML cells are still not clearly understood. Moreover, it is still not known the importance of cell-cell interaction in chemoprotection and whether interaction with stromal cells could lead to emergence of chemoresistant CML cells at physiologically relevant dosage of IM. In our study, we sought to identify the stroma dependent aberrant molecular signaling pathways in CML cells that play a crucial role in CML chemoprotection and emergence of chemoresistance.

## Results

### Stromal cells chemoprotect CML cells through direct cell-cell contact

**Figure 1:**
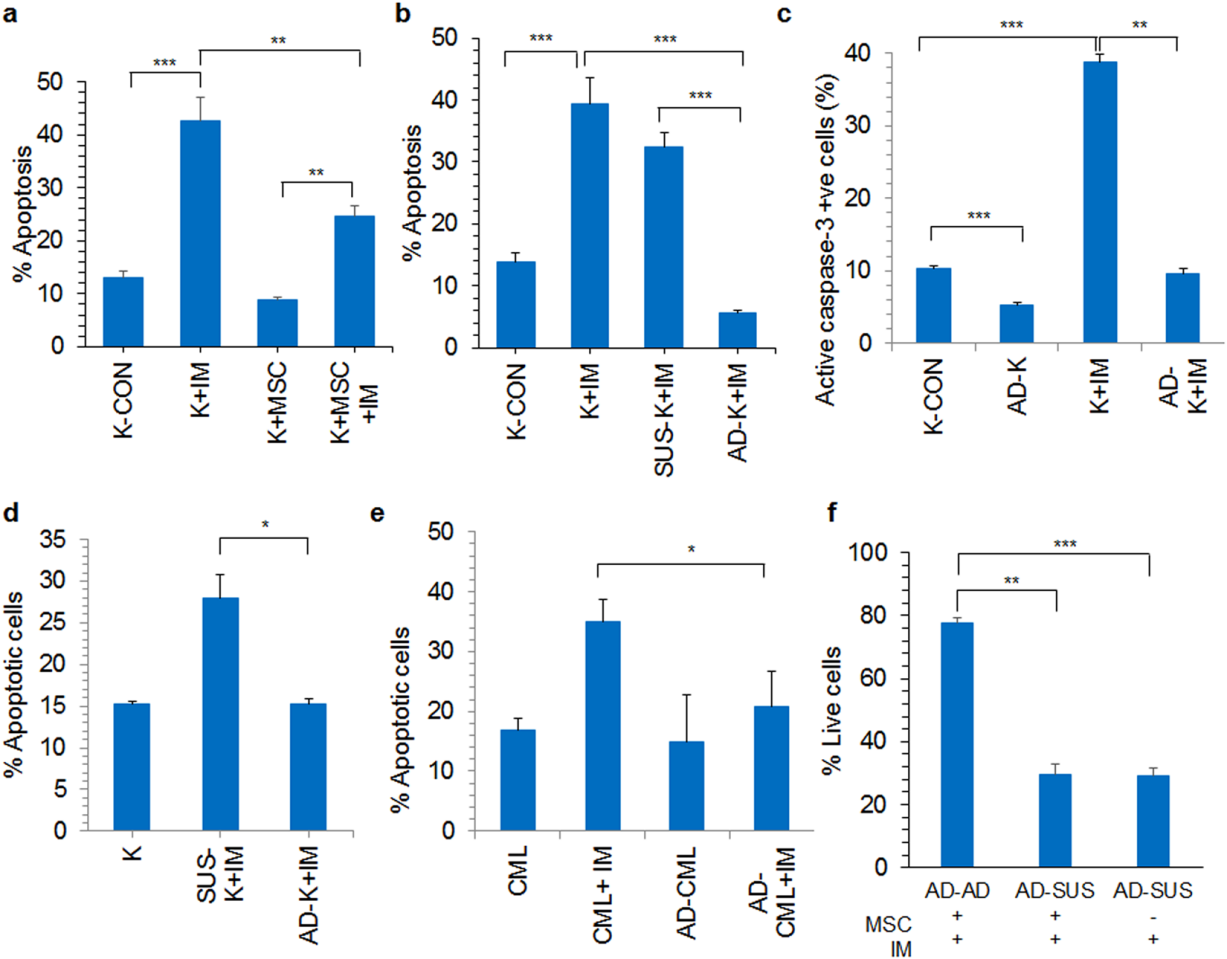
Stroma adherent CML cells were chemoprotected from IM treatment. (**a**) K562 CML cells were co-cultured with (K + MSC) or without (K) stromal cells and treated with (+IM, 10 µM) or without IM for 48 hours and cell death was analyzed by annexin-V/PI staining. (**b**) The stroma co-cultured K562 cells were treated with IM for 48 hours and apoptosis was analyzed in suspension fraction (SUS-K) and adherent fractions (AD-K) separately. (**c**) K562 cells co-cultured with and without stromal cells were treated with IM for 48 hours and percentage of cells positive for active caspase-3 was analyzed by flow cytometry. Stroma adherent K562 cells (AD-K) were compared with control K562 cells (K) treated with or without IM. (**d**) K562 cells were co-cultured with stromal cells and the suspension fraction (SUS-K) was transferred to a new stromal layer leaving AD-K undisturbed, IM was added to the co-culture containing either AD-K or SUS-K only and apoptosis was analyzed after 48 hours of IM treatment. (**e**) CML patient derived bone marrow mononuclear cells at the chronic phase were co-cultured with CML derived stromal cells (AD-CML). The control and the co-cultured cells were treated with IM and apoptosis percentage was analyzed as indicated. (**f**) K562 cells were co-cultured with stromal cells and the suspension fraction was discarded. The adherent cells were treated with IM and the resulting AD-K cells were transferred into a fresh stromal layer (AD-AD, AD-SUS) or a stroma free culture (AD-SUS) and treated with IM. The percentage of live cells in each of the fractions was determined by annexin-V/PI staining. Pictorial representation of the experiment is shown in supplementary figure 2. *p < 0.05, **p < 0.005, ***p < 0.0005, n ≥ 3.

When CML (K562) cells were cultured in contact with the stromal cells in the microenvironment, CML cells were protected from the apoptotic effect of chemotherapeutic agent imatinib mesylate (IM)(Fig. 1a), as reported also by others^16^. During culture with stromal cells, we observed that a fraction of CML cells were adherent to the stroma. We separated the co-cultured K562 cells into stroma adherent (AD-K) and stroma non-adherent suspension (SUS-K) fractions and apoptosis percentage was determined during IM treatment and compared with K562 cells cultured without stromal cells (K-CON). Whereas the SUS-K cells had similar apoptosis percentage as control K562 cells, AD-K had significantly lower IM induced apoptosis (Fig. 1b). Since IM induces cell death through activation of caspase-3^17^, the percentage of active caspase-3 positive cells was determined in AD-K and SUS-K cells. There was a significantly high percentage of active caspase-3 positive cells in IM treated SUS-K cells (39.8%), whereas, it was similar to the untreated controls (9.5%) in IM treated AD-K (8.9%) cells (Fig. 1c), confirming that the K562 cells adherent to the stroma layer were chemoprotected. To understand whether the decreased apoptosis in IM treated AD-K cells was due to low drug availability to the adherent cells, SUS-K cells were removed from the co-culture and IM was added to AD-K cells in co-culture. After IM treatment, both adherent and non-adherent K562 cells were pooled together from the co-culture and tested for apoptosis percentage. A significantly reduced apoptosis was observed in AD-K cells treated with IM in the absence of SUS-K cells during co-culture (Fig. 1d). Similar results were obtained in case of primary chronic phase CML cells. Before initiating IM treatment, the suspension cells were removed from the co-culture and only stroma adherent primary CML cells were treated. When both adherent and non-adherent fractions of CML cells were analyzed for apoptosis, we found a significantly reduced apoptosis in IM treated stroma adherent CML cells compared to the control cells (Fig. 1e). To test whether AD-K cells could be made susceptible to IM treatment by disrupting cell-cell adhesion, IM treated AD-K cells were transferred to a stroma free culture and when treated with IM, the percentage of apoptosis was similar to the control cells cultured in stroma free condition. However, the cells that were maintained as stroma adherent, showed significantly low cell death and high live cell percentage (Fig. 1f) pointing to the fact that cell-cell adhesion was important for chemoprotection of CML cells from chemo drug induced cell death.

**Figure 2:**
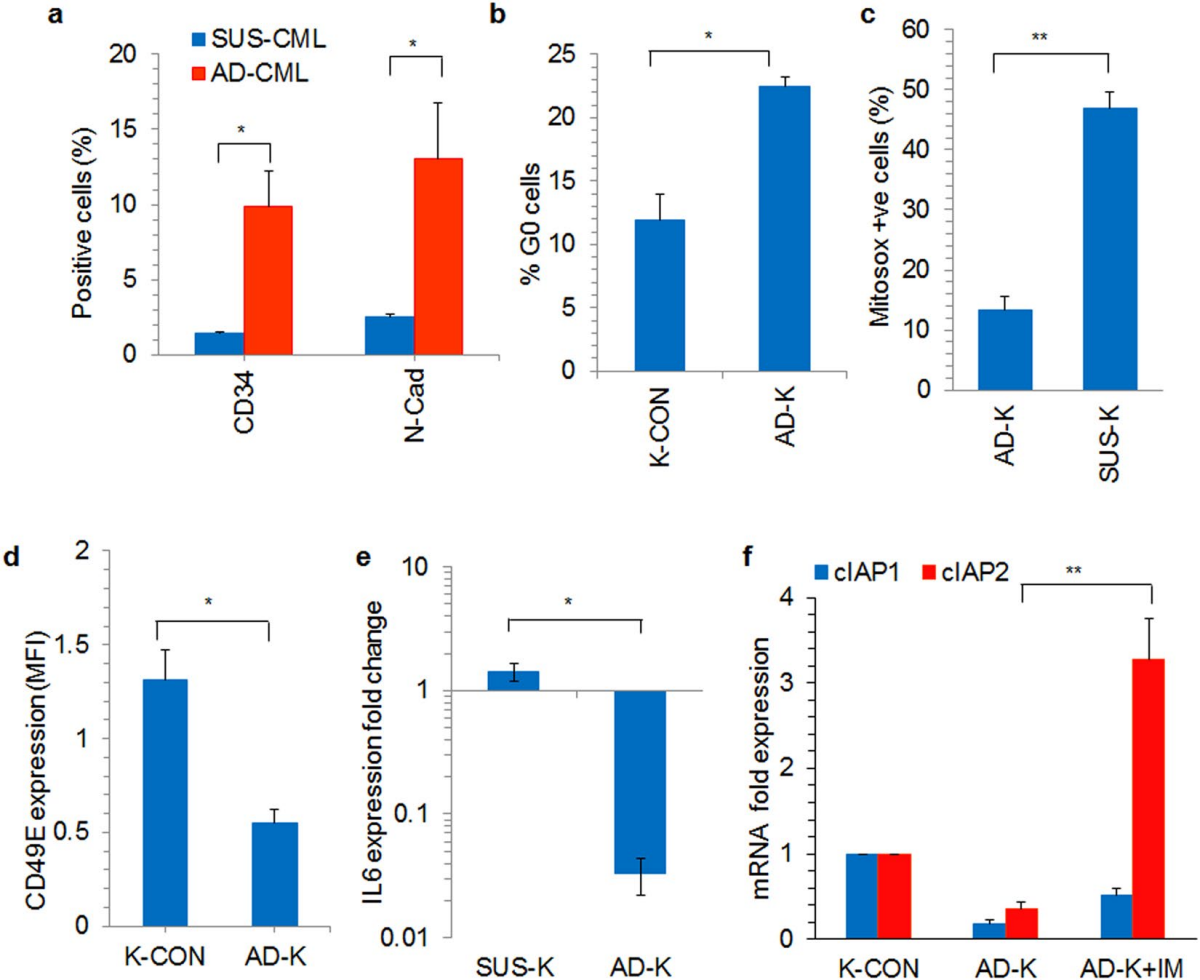
(**a**) Stroma adherent CML primary cells were separated from the suspension cells and the percentage of CD34 and N-cadherin positive cells were analyzed in both suspension fraction (SUS-CML) and stroma adherent fraction (AD-CML). Stroma adherent (AD-K) and control (K-CON) K562 cells were analyzed for their (**b**) cell cycle G0 status by Ki67 staining (**c**) ROS production using mitosox mitochondrial ROS detection stain and (**d**) CD49E cell surface expression. (**e**,**f**) RNA was extracted and reverse transcribed into cDNA from stroma adherent (AD-K) and suspension (SUS-K) fractions of co-cultured K562 cells. (**e**) IL6 and (f) cIAP1 and cIAP2 transcript levels were determined through real-time PCR. The expression values were normalized to GAPDH levels in the respective samples. *p < 0.05, **p < 0.005, n ≥ 3.

Additionally, when primary CML cells were co-cultured with stromal cells, the adherent CML cells had seven fold higher percentage of CD34 positive cells and five-fold higher N-Cadherin positive cells compared to the suspension cells (Fig. 2a). AD-K cells had higher percentage of cells in G0 stage of the cell cycle compared to the suspension cells as determined by Ki67 staining (Fig. 2b). AD-K cells also had significantly lower mitochondrial ROS production compared to SUS-K cells (Fig. 2c). AD-K cells had reduced cell surface expression of integrin CD49E (Fig. 2d), lower IL6 transcript levels (Fig. 2e) and higher cIAP2 expression levels during IM treatment compared to the SUS-K cells (Fig. 2f).

### Mediators of CML-**stroma** adherence

**Figure 3:**
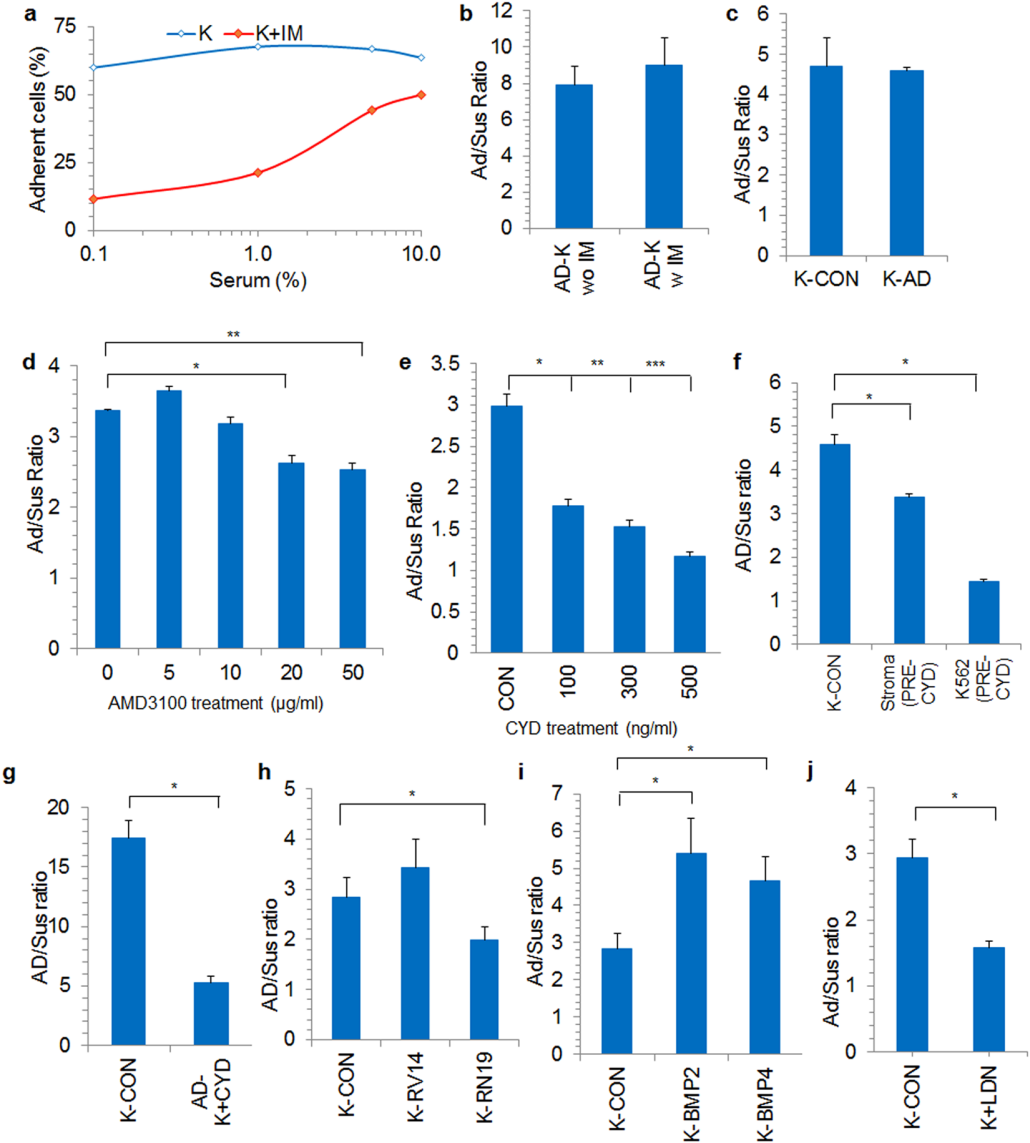
(**a**) Percentage of stroma adherent cells were calculated in IM treated (K + IM) and untreated (K) cells seeded at different serum concentrations. (**b**) 48 hrs adherent K562 cells were treated with IM for 24hrs (AD-K w IM) and AD/SUS cells ratio was compared with adherent cells not treated with IM (AD-K wo IM). Pictorial representation of the experiment is shown in supplementary figure 3. (**c**) 48 hrs adherent K562 cells (AD-K) were seeded on a fresh stromal layer and Ad/Sus cells ratio was compared with control K562 cells (K-CON). Pictorial representation of the experiment is shown in supplementary figure 3. (**d**,**e**) K562 cells were seeded in stroma co-culture and the cells were treated with (**d**) indicated concentrations of CXCR4 inhibitor AMD3100 (µg/ml) or (**e**) indicated concentrations of cytochalasin D (CYD, ng/ml). The adherent and suspension cell fractions were collected, microscopically counted and the adherent versus suspension ratio (Ad/Sus) was calculated. Ad/Sus ratio was calculated for K562 (**f**) seeded either on CYD pre-treated MSC (Stroma PRE-CYD) or untreated MSC but pre-treated with CYD (K562 PRE-CYD) (**g**) CYD was added to the co-culture containing adherent K562 cells (AD-K + CYD). The Ad/Sus ratio was also calculated for co-cultured K562 cells lentivirally transduced with (**h**) constitutively active RHOA (K-RV14), dominant negative RHOA (K-RN19) plasmids, (**i**) overexpressing BMP2 (K-BMP2), BMP4 (K-BMP4) or (**j**) treated with BMP inhibitor LDN193189 (K + LDN). *p < 0.05, **p < 0.005, ***p < 0.0005, n ≥ 3.

From initial experiments, we concluded that adhesion plays an important role in CML chemoprotection. To understand the molecules that mediate the adhesion of leukemic cells to the stromal layer, first, we sought to find out whether IM treatment in itself could increase leukemia cells adhesion to the stroma. IM treatment at different serum concentrations did not facilitate cell-cell interaction, rather it inhibited leukemic cell adhesion to the stromal cells (Fig. 3a). Moreover, when AD-K cells were treated with IM, it did not lead to the detachment of the stroma adherent cells (Fig. 3b). When AD-K cells were transferred to a fresh stromal layer, they did not possess a higher stroma adherence but were similar to the control cells (Fig. 3c). However, treatment with CXCR4 inhibitor AMD3100 (Fig. 3d) and disruption of actin cytoskeleton with cytochalasin D (CYD) treatment abrogated CML-stromal interaction. CYD reduced the K562 cell adhesion in a dose dependent manner (Fig. 3e). Pretreatment of either K562 or stromal cells with CYD was equally effective in reducing K562 cell adhesion to stroma (Fig. 3f). Interestingly, when CYD was added to the culture with a high percentage of already existing stroma adherent cells, it significantly disrupted the cell-cell interaction (Fig. 3g). Since actin was found to be important for cell-cell adhesion, we studied RhoA signaling that regulates actin cytoskeleton. Downregulation of RHOA GTPase through RHOAN19 expression^18^ significantly decreased K562 adhesion to the stromal cells (Fig. 3h). Since BMPs were shown to regulate CML cell survival^19^, we determined the stroma adherence of BMP2 and BMP4 overexpressing K562 cells. BMP2 and BMP4 overexpression significantly increased K562 cell adhesion to the stromal cells (Fig. 3i). Conversely, inhibition of BMP signaling by LDN193189 treatment significantly reduced K562 cell adhesion to the stroma (Fig. 3j).

### ERK1/2 and BMP signaling pathways are affected in stroma adherent K562 cells

**Figure 4:**
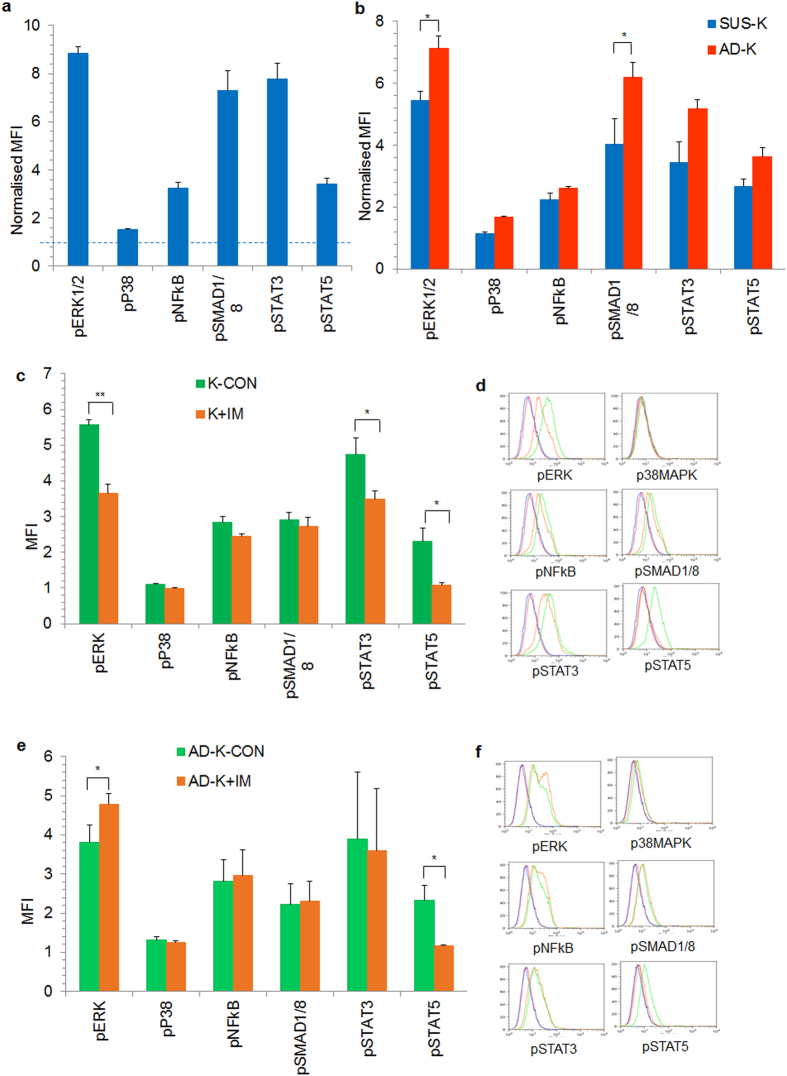
**S**troma adherent K562 cells modified key signaling pathways during IM treatment. (**a**) The active signaling pathways in K562 cells were determined by analyzing the phosphorylation status of ERK1/2 MAPK, p38 MAPK, NFκB, SMAD1/8, STAT3 and STAT5 by phospho flowcytometry. (**b**) Stroma co-cultured K562 cells were separated into SUS-K, AD-K cells and phospho protein levels of indicated signaling molecules were analyzed. (**c**) Control K562 cells cultured without the stromal layer treated with (K + IM) or without (K) IM were analyzed for the indicated phospho protein levels. (**d**) Flow cytometric histogram representing the phospho protein analysis in control K562 cells treated with and without IM as indicated in (**c**). Red line represents the isotype control cells for control cells (K) and green line shows the stained (K) cells. Blue line represents the K + IM isotype control and orange line represents the stained K + IM condition. (**e**) Stroma adherent K562 cells (AD-K) were treated without (AD-K-CON) or with IM (AD-K + IM) and the phospho proteins levels of the indicated signaling molecules were analyzed. (**f**) Flow cytometric histograms representing the phospho protein analysis in AD-K cells treated with and without IM as indicated in (**e**). Red line represents the AD-K isotype control and green line shows the stained AD-K cells. Blue line represents the AD-K + IM isotype control and orange line represents the stained AD-K + IM condition. The expression levels were normalized against respective isotype controls. Values are represented as mean (Geometric mean) fluorescent intensity (MFI). *p < 0.05, n ≥ 5.

We studied whether the AD-K cells have activated key signaling pathways for chemoprotection during IM treatment, which could be utilized as targets for therapy. Phosphorylation analysis of several signaling molecules showed that STAT3, STAT5, NFκB, ERK1/2 MAPK, SMAD1/8 signaling pathways were active in K562 cells (Fig. 4a). While levels of pNFκB was similar in both AD-K and SUS-K cells, we found significantly high levels of pERK1/2 MAPK, pSMAD1/8 in AD-K cells (Fig. 4b) and although not significant, pSTAT3 and pSTAT5 levels were higher in AD-K compared to SUS-K cells (Fig. 4b). IM treatment led to reduction in levels of pSTAT3, pSTAT5 and pERK1/2 in control K562 cells (Fig. 4c,d), indicating inhibition of BCR-ABL activity. In AD-K cells on the other hand, even though pSTAT5 was downregulated during IM treatment, surprisingly, pERK1/2 levels were significantly upregulated. pSTAT3 or pSMAD1/8 protein levels were unaffected during IM treatment (Fig. 4e,f). Hence, we concluded that even though abrogation of BCR-ABL activity downregulated pERK1/2 levels in control K562 cells, adhesion to stroma upregulated its levels in the presence of IM. Moreover, pSMAD1/8 level was unaffected by IM treatment but was upregulated by stromal interactions (Fig. 4b).

### Targeting ERK1/2 and BMP signaling pathways and adhesion mediators in stroma adherent K562 cells

**Figure 5:**
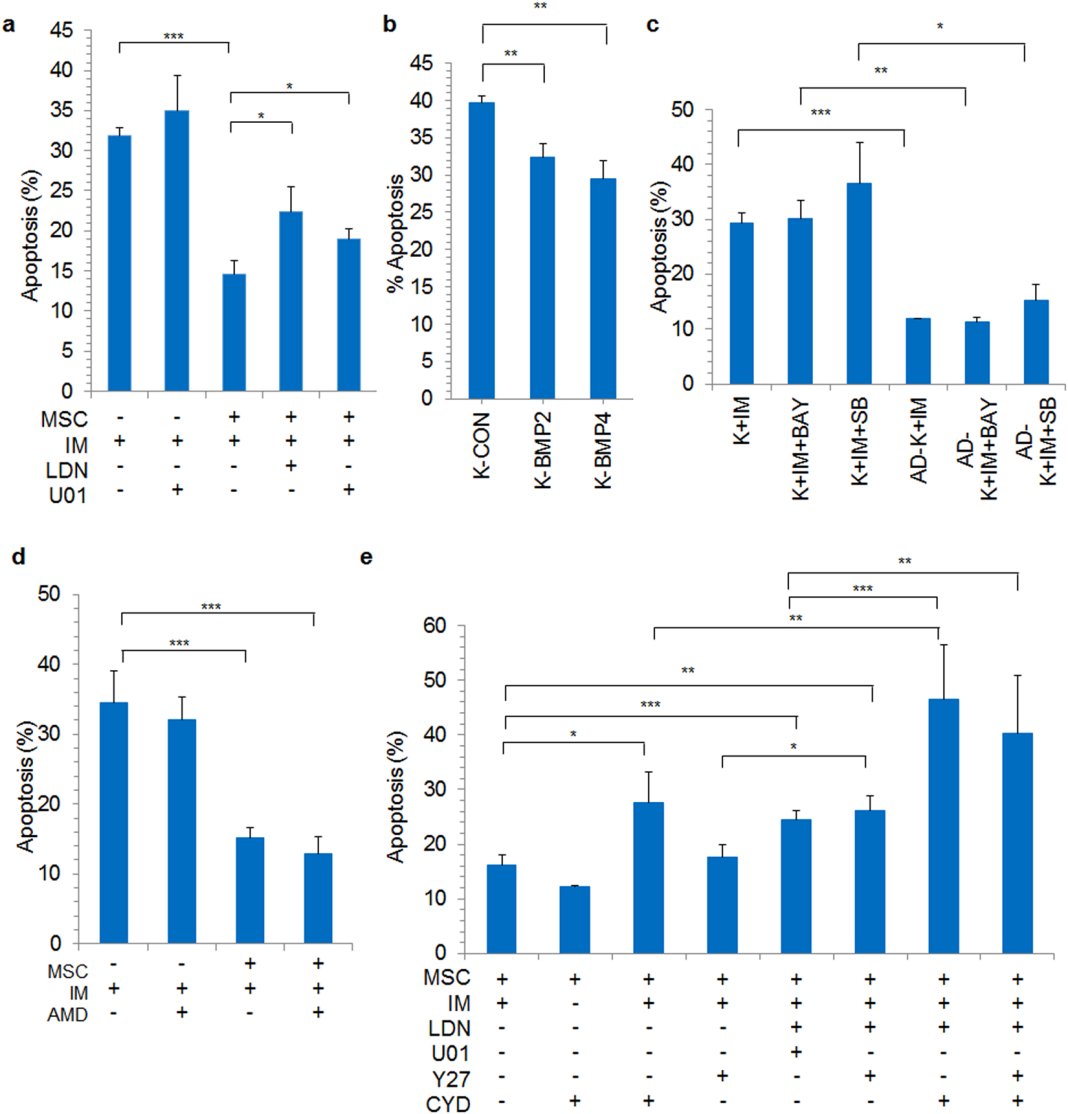
(**a**) K562 cells adherent to stromal cells (MSC+) were treated with BMP inhibitor LDN193189 (LDN, 3 μM), ERK inhibitor U0126 (U01, 10 μM) and apoptosis percentage was analyzed. (**b**) Apoptosis percentage was analyzed in K562 cells overexpressing BMP2, BMP4 and treated with IM (10 µM for 48 hrs). Apoptosis percentage was analyzed in control (K) or adherent (AD-K) K562 cells treated (**c**) with NFκB inhibitor Bay 11-7082 (BAY) or P38 MAPK inhibitor SB203580 (SB) in the presence of IM for 48 hours, (**d**) CXCR4 inhibitor AMD3100 (AMD, 50 μg/ml), (**e**) BMP inhibitor LDN193189 (LDN, 3 μM), ERK inhibitor U0126 (U01, 10 μM), ROCK inhibitor Y27632 (Y27, 20 μM) and cytochalasin D (CYD, 0.3 μg/ml) along with IM (10 μM). Apoptosis percentage was determined after 48 hours of treatment. *p < 0.05, **p < 0.005, ***p < 0.0005, n = 3–5.

We utilized small molecule inhibitors against specific signaling pathways as well as adhesion mediators which were identified in previous sections, to sensitize AD-K cells to IM treatment. K562 CML cells were added to stromal layer and after 48 hrs, suspension cells were removed. IM was added along with specific inhibitors and after 48 hrs apoptosis was analyzed. Since, pERK1/2 levels were upregulated in AD-K cells upon IM treatment; first we tested ERK1/2 signaling inhibitor U0126. U0126 (10 μM) treatment sensitized AD-K cells to IM, thus increasing their apoptosis cell percentage but not in the control cells (Fig. 5a). Similar increase in apoptosis was observed when AD-K cells were treated with BMP signaling inhibitor LDN193189 (3 μM), confirming the role of these pathways in chemoprotection of CML cells against IM (Fig. 5a). Conversely, when BMP2 and BMP4 overexpressing cells were treated with IM, we observed significantly reduced apoptosis (Fig. 5b). Moreover, inhibiting NFκB and P38 MAPK signaling with BAY 11-7082 and SB203580 respectively did not sensitize AD-K cells to IM treatment (Fig. 5c). Inhibition of CXCR4 signaling with AMD3100 in the presence of IM did not have any effect on apoptosis of AD-K cells (Fig. 5d) although it disrupted cell-cell interaction of leukemic cells with the stroma (Fig. 3d). However, inhibition of actin cytoskeleton with CYD significantly increased the apoptosis percentage in AD-K cells (Fig. 5e) in addition to inhibiting the cell-cell interaction of leukemic cells with stroma (Fig. 3g).

To achieve maximum sensitization of chemoprotected AD-K cells to IM, we tested combinations of pathway inhibitors. Combining U0126 with LDN193189 was more effective in chemosensitizing AD-K cells than treatment with individual inhibitors. ROCK inhibitor Y27632 which abrogates RHOA signaling as well as CYD which inhibits actin polymerization were more effective when added with LDN193189 in inducing apoptosis in AD-K cells in the presence of IM (Fig. 5e). Thus, inhibition of ERK1/2 and BMP signaling alone or in combination with inhibitors of adhesion mediators was effective in chemosensitizing AD-K cells to IM.

### Derivation of chemoresistant K562 CML cells through stromal co-culture

**Figure 6:**
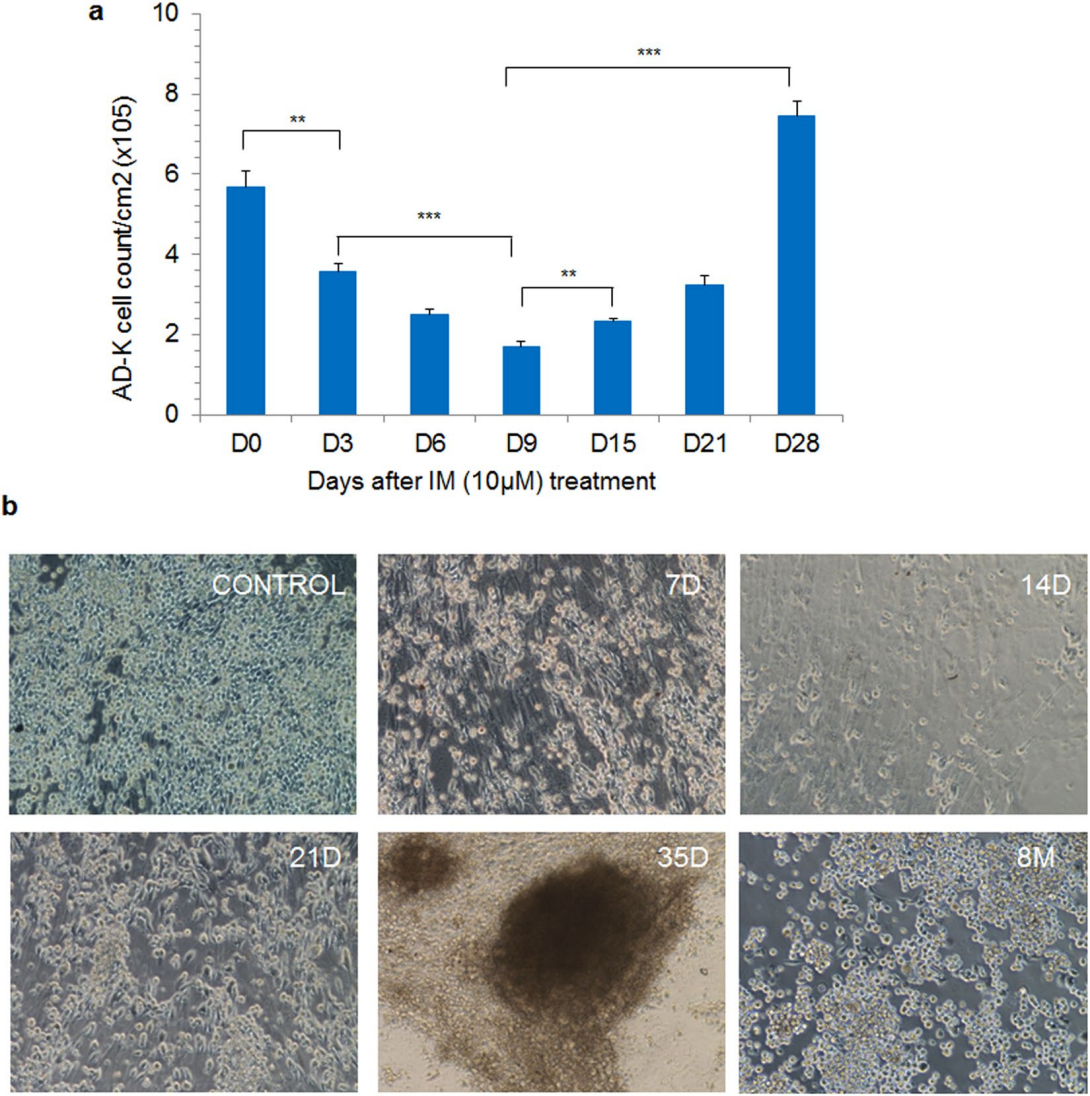
(**a**) K562 cells were co-cultured with stromal cells in the presence of IM (10 μM) and the number of stroma adherent cells (AD-K) was counted at the indicated time intervals. (**b**) Microscopic images showing stroma co-cultured K562 cells (AD-K) treated with IM for 7 days (7D), 14 days (14D), 21 days (21D) or 35 days (35D) or 8 months (8 M) with IM (10 µM). Magnification 10x. **p < 0.005, ***p < 0.0005, n = 3–5.

We studied whether the short-term chemoprotection observed in AD-K cells can lead to the development of stroma independent chemoresistance. For this, we co-cultured K562 cells with stromal layer in the presence of IM for longer durations (10–12 weeks). Upon continuous treatment with IM, majority of AD-K cells underwent cell death and AD-K cell number decreased steadily until 2–3 weeks (Fig. 6a). However, after 2–3 weeks, the persistent adherent cells grew as localized cell colonies attached to the stromal layer (Fig. 6b). These cell colonies continued to grow on multiple sites and we observed an increase in AD-K cell number until 4–5 weeks (Fig. 6a). The co-culture system could be maintained for longer durations (longest cultures were maintained for 8 months on the same stromal layer) until chemoresistant cells emerged (Fig. 6b).

**Figure 7:**
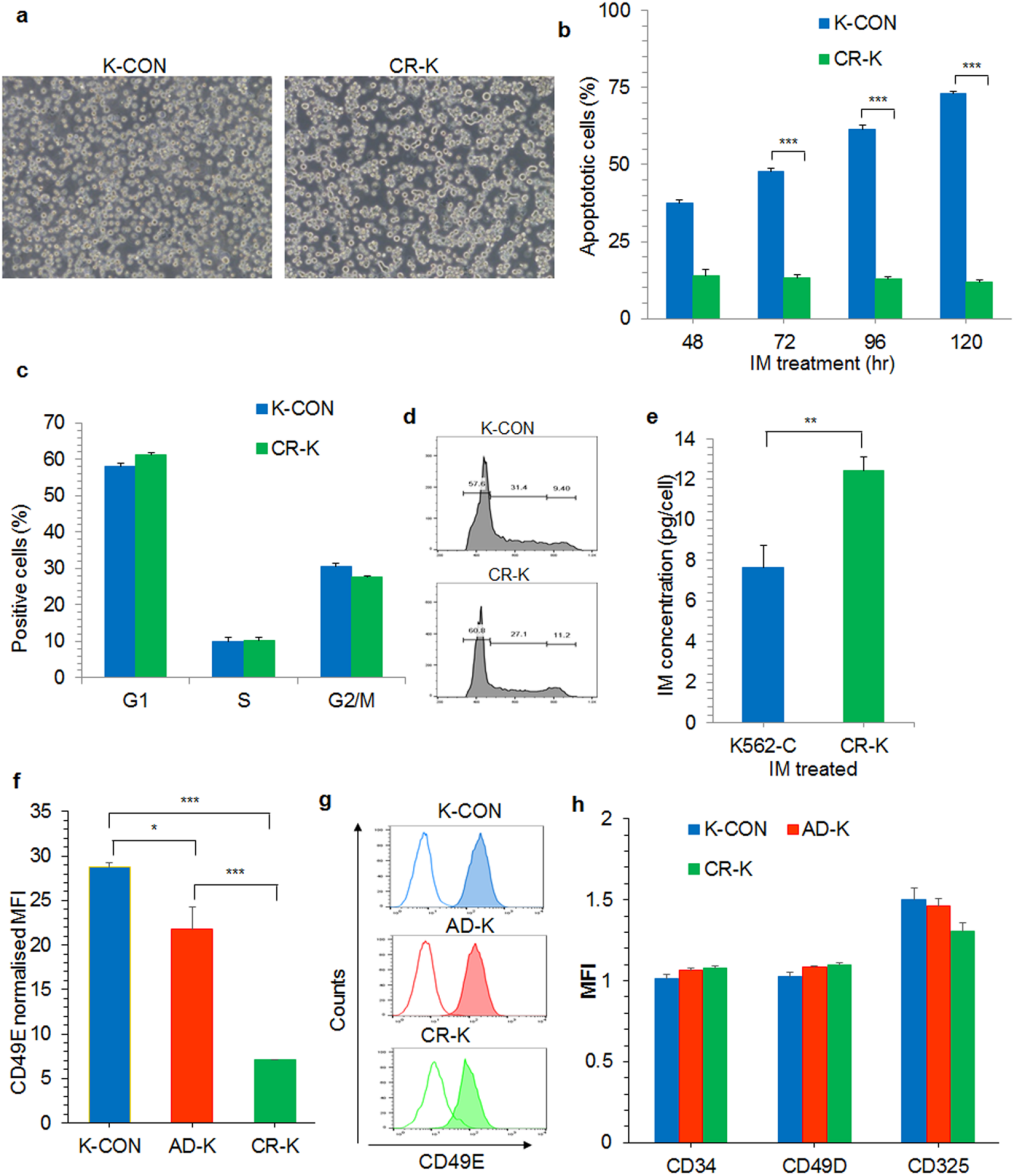
The stroma adherent K562 cells in the co-culture during continuous treatment with IM were transferred to a stroma free culture after 10–12 weeks and treated with IM. (**a**) Microscopic image showing control K562 cells (K-CON) or IM treated chemoresistance K562 cells (CR-K). (**b**) The percentage of apoptosis was analyzed in IM treated control K562 cells (K-CON) and AD-K converted into chemoresistant K562 (CR-K) cells at different time intervals as indicated. (**c**) Percentage of cells in different stages of cell cycle in control K562 cells (K-CON) and chemoresistant K562 cells growing in the presence of IM (CR-K). (**d**) Histogram showing cell cycle profile of K-CON and CR-K as presented in (**c**). (**e**) Intracellular IM concentration was measured in IM treated control (K-CON) and chemoresistant (CR-K) K562 cells. (**f**,**g**) The cell surface antigen expression was analyzed in control (K-CON), long-term stroma adherent (AD-K) K562 cells cultured in the presence of IM for 6 weeks and chemoresistant (CR-K) K562 cells cultured in the presence of IM. The indicated cells types were (**f**) analyzed for the expression of CD49E and (**g**) the representative histograms showing CD49E expression in K-CON, AD-K and CR-K cells. The unfilled lines were the isotype controls and the filled histograms represent the stained sample. (**h**) Cell surface expression levels of CD34, CD49D and CD325 (N-Cadherin) were also analyzed in K-CON, AD-K and CR-K cells. *p < 0.05, **p < 0.005, ***p < 0.0005, n = 3.

After 10–12 weeks of IM treatment in the co-culture system, AD-K cells were transferred to a stroma free culture and interestingly, these cells proliferated in stroma free culture (Fig. 7a) and were termed as chemoresistant K562 (CR-K) cells. These CR-K cells were thereafter cultured continuously in the presence of IM unless otherwise mentioned. CR-K cells exhibited only minimal levels of apoptosis, whereas more than 90% of the control K562 cells underwent apoptosis after 96 hrs of IM treatment (Fig. 7b). CR-K cells had cell cycle profile similar to that of control K562 cells (Fig. 7c,d). However, when the intracellular levels of IM were tested, CR-K cells had significantly higher levels of intracellular IM than IM treated control K562 cells (Fig. 7e), indicating that the acquired chemoresistance was not due to drug efflux in CR-K cells. Chemoresistant cells underwent changes in their cell surface marker expression where we observed a significant downregulation in CD49E expression in both long-term AD-K and CR-K cells compared to control K562 cells (Fig. 7f,g) whereas CD34, CD49D and CD325 cell surface expression were not altered (Fig. 7h).

**Figure 8:**
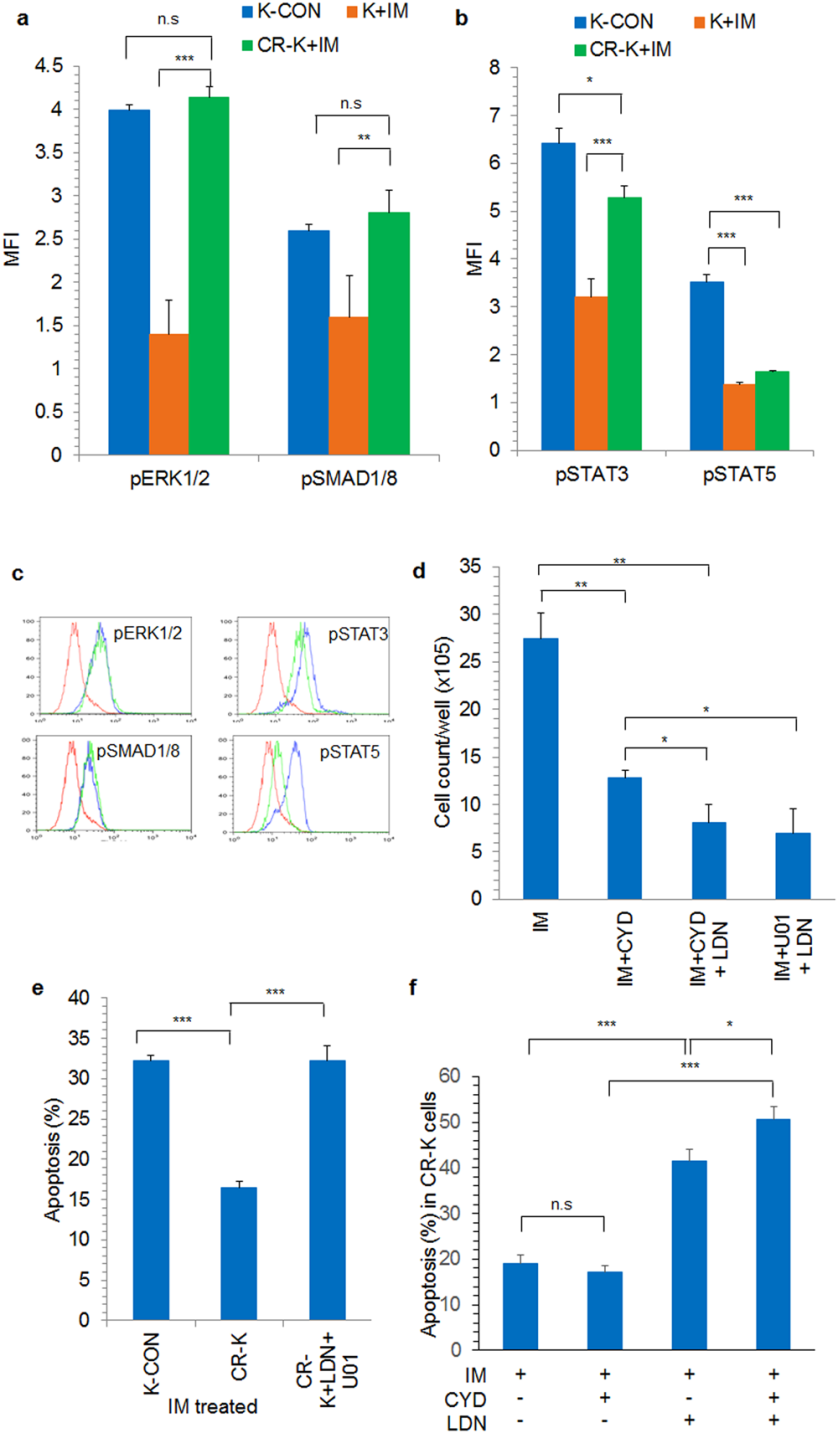
Chemoresistant K562 cells have active ERK and SMAD signaling during IM treatment. Phospho protein levels of (**a**) ERK1/2, SMAD1/8 (**b**) STAT3 and STAT5 were determined in control (K-CON), control K562 treated with IM (K + IM) and chemoresistant K562 cells grown in the presence of IM (CR-K + IM). The expression levels were normalized against the isotype control. (**c**) Representative flow cytometric histograms showing the expression of ERK1/2, SMAD1/8, STAT3 and STAT5 in control K562 cells (blue line) and chemoresistant K562 cells (green line). The red line represents the isotype control. (**d**) Long-term stroma adherent IM treated K562 cells were treated with combinations of inhibitors along with IM as indicated for 2 weeks and the number of live adherent cells were counted. (**e**) Stroma independent chemoresistant K562 cells (CR-K) were treated with combinations of LDN193189 and U0126 (CR-K + LDN + U01) along with IM and the percentage apoptosis was compared with IM treated control K562 cells (K-CON). (**f**) CR-K cells were treated with CYD, LDN or combination of CYD and LDN and apoptosis percentage was analyzed. CYD-Cytochalasin D; LDN-LDN193189; U01-U0126; IM-Imatinib. *p < 0.05, **p < 0.005, ***p < 0.0005, n = 3–5.

During co-culture, we found that AD-K cells had higher active levels of ERK and BMP signaling and inhibiting these pathways chemosensitized AD-K to IM (Fig. 5a,b). Similarly, in IM treated CR-K cells, higher phospho levels of ERK1/2 and SMAD were maintained, whereas in control K562 cells it was significantly reduced upon IM treatment, suggesting the involvement of ERK and BMP pathways in regulating IM resistance (Fig. 8a–c). Moreover, after 5 weeks of co-culture with IM, when persistent AD-K cells were treated with CYD, LDN193189 and U0126, a significant reduction in adherent cell population was observed (Fig. 8d). Importantly, treatment with BMP and ERK inhibitors sensitized the CR-K cells to IM treatment resulting in a significant increase in the apoptotic cells percentage (Fig. 8e). Although, these cells proliferated in the presence of IM, treatment with the pathway inhibitors (ERK and BMP) for 48 hours was sufficient to induce significant apoptosis (Fig. 8e). Moreover, CYD had synergistic effect in inducing apoptosis in CR-K cells when added along with LDN193189 but not alone in the presence of IM (Fig. 8f).

Thus, stromal interaction during IM treatment chemoprotected CML cells and on long term treatment, chemoresistant cells developed with aberrant signaling pathways. Targeting the adhesion mediators, ERK and BMP signaling pathways during initial treatment or using a combinatorial approach after chemoresistance development in the CML patients will more likely result in complete eradication of leukemic cells.

## Discussion

Leukemic cells interact with stromal cells in the bone marrow via direct cell contact and reside in contact with stromal cells such as the osteoblasts^20^. This direct interaction between the stromal and the leukemic cells has been hypothesized as a major contributor in the persistence of leukemic cells in CML patients following TKI treatment^15^. In agreement with this hypothesis, in our study, we found that the cell-cell contact between CML cells and the stromal cells chemoprotected CML cells during IM treatment but IM treatment did not contribute to the enhanced adhesion of CML cells to the stromal layer. Moreover, a short-term interaction with the stromal cells was sufficient for chemoprotection but not induction of chemoresistance in CML cells, because when the adherent cells were transferred to stroma free culture, they became susceptible to apoptosis induction by IM. This also provides a strategy that if the stroma adherent cells could be brought into suspension at the early stages of IM therapy, then majority of the leukemic cells could be eliminated. However, if the leukemic cells remain adhered to the stromal cells throughout the chemotherapeutic drug treatment in the patients, it might give rise to chemoresistant leukemic cells.

As reported by others^16^, stroma adherent CML cells were found to be more quiescent compared to suspension CML cells. Since TKIs induce cell death in BCR-ABL + cells by blocking their proliferation, TKIs are not equally effective on non-cycling or quiescent leukemic cells. Leukemic cells have higher levels of intrinsic reactive oxygen species (ROS) and BCR-ABL expression was reported to increase ROS levels in hematopoietic cells. However, LT-HSC reside in low oxygenic endosteal niche in the BM which limits ROS levels in LT-HSC and maintain them in undifferentiated state^21^. Similar to what was reported in AML cells^22^, AD-K cells had low ROS levels compared to CML cells in suspension. This suggests that the stromal cells might reduce the metabolic stress in the leukemic cells and keep them in a non-cycling quiescent state which protects leukemic cells from drug induced cell death. Notably, our results also showed that stroma adherent CML cells were enriched for stem cells expressing CD34 and N-Cadherin. Increased levels of anti-apoptotic gene cIAP2 levels might represent one of the mechanisms through which AD-K cells resist IM induced apoptosis as IAPs have been implicated in cancer cell survival^23^.

We found that adhesion to stroma could be reduced by inhibiting CXCR4 with AMD3100 but was not sufficient to chemosensitize AD-K cells to IM. However, Vianello *et al*. found increased apoptosis upon CXCR4 inhibition during IM treatment^24^ and Zepeda-Moreno *et al*. did not identify any change in cell adhesion^25^. These discrepancies might be due to the difference in experimental setup where we specifically assayed AD-K cells which were more prone to develop into chemoresistant cells. Actin cytoskeleton is involved in adhesion of different cell types to extracellular surface and also involved in integrin related signal transduction^26^. In the present study, disrupting actin polymerization by CYD treatment resulted in significant reduction of K562 cell adhesion to the stromal layer, which were effectively eliminated by IM treatment. Trendowski *et al*. reported that addition of CYD and (cytochalasin B) CYB showed synergistic effect with doxorubicin on chemoresistant murine leukemia cells^27^. When combined with IM, CYD at non-toxic concentration of 0.3 µg/ml resulted in increased apoptosis of adherent K562 cells. Similarly, inhibition of RHOA which modifies actin^28, 29^ also resulted in reduced CML cell adhesion to the stroma.

Phospho flow cytometric analysis showed that ERK1/2 MAPK, NFκB, SMAD1/8, STAT3 and STAT5 signaling pathways were active in K562 cells. However, AD-K cells had higher levels of pERK1/2 MAPK and pSMAD1/8 compared to suspension K562 cells. IM treatment resulted in downregulation of pERK1/2, pSTAT3 and pSTAT5 levels in K562 cells in suspension, whereas in AD-K cells, pERK1/2 levels were upregulated. Our results suggest that ERK1/2 MAPK pathway was activated in CML cells through stromal interaction and this effect was independent of BCR-ABL kinase activity. A potential role of ERK1/2 MAPK pathway in survival^30^ and chemoresistance of CML was highlighted in studies by others as well. Increased pERK1/2 levels was reported in CD34 + CML cells upon IM treatment^31^. Similarly, induction of FGF2 mediated chemoresistance in K562 cells against IM was also associated with high levels of pERK1/2^13^. Our data is in agreement with the report by others that ERK1/2 is activated by cell adhesion leading to cell survival and inhibition of which leads to apoptosis in CML as well as multiple myeloma cells^32, 33^. Higher levels of BMP2 and BMP4 in CML BM microenvironment were reported to be responsible for maintenance and expansion of LSC and myeloid progenitors^19^. BMP-SMAD signaling are BCR-ABL independent as pSMAD1/8 levels were unaffected by IM treatment in CML cells. In our study, BMP2 and BMP4 transduced K562 cells showed significantly higher adhesion to the stromal layer and reduced susceptibility to IM induced apoptosis. Inhibition of ERK1/2 and BMP-SMAD signaling by treating the cells with U0126 and LDN193189 respectively, significantly increased the chemosensitivity of AD-K cells to IM.

Finally, an important aspect of our study was the method employed to generate chemoresistant CML cells that closely resembles the physiological condition. In earlier studies, chemoresistant K562 cells were developed by continuous exposure to low concentrations of IM. Additionally, the modes used for development of chemoresistant cells were either exposure to growth factors^13^ or stepwise increase in IM concentration until resistant K562 cells appeared^33^. However, in CML patients, the leukemic cells interact with the stromal cells and are continuously exposed to high dose of IM. Hence in our study, a co-culture system was established where stroma adherent K562 cells were continually exposed to physiological concentrations of IM until the chemoresistant K562 cells emerged. AD-K562 cells underwent apoptosis after continuous IM treatment, however, persistent AD-K cells started proliferating, which when transferred to a stroma free culture continued to divide confirming the acquisition of stroma independent IM resistance. Surprisingly, treatment of AD-K in long-term culture with combinations of U0126, LDN193189 and CYD, in presence of IM abrogated the development of chemoresistant cells. Moreover, treatment with U0126 and LDN193189 or a combination of CYD and LDN193189 sensitized CR-K to IM induced cell death.

**Figure 9:**
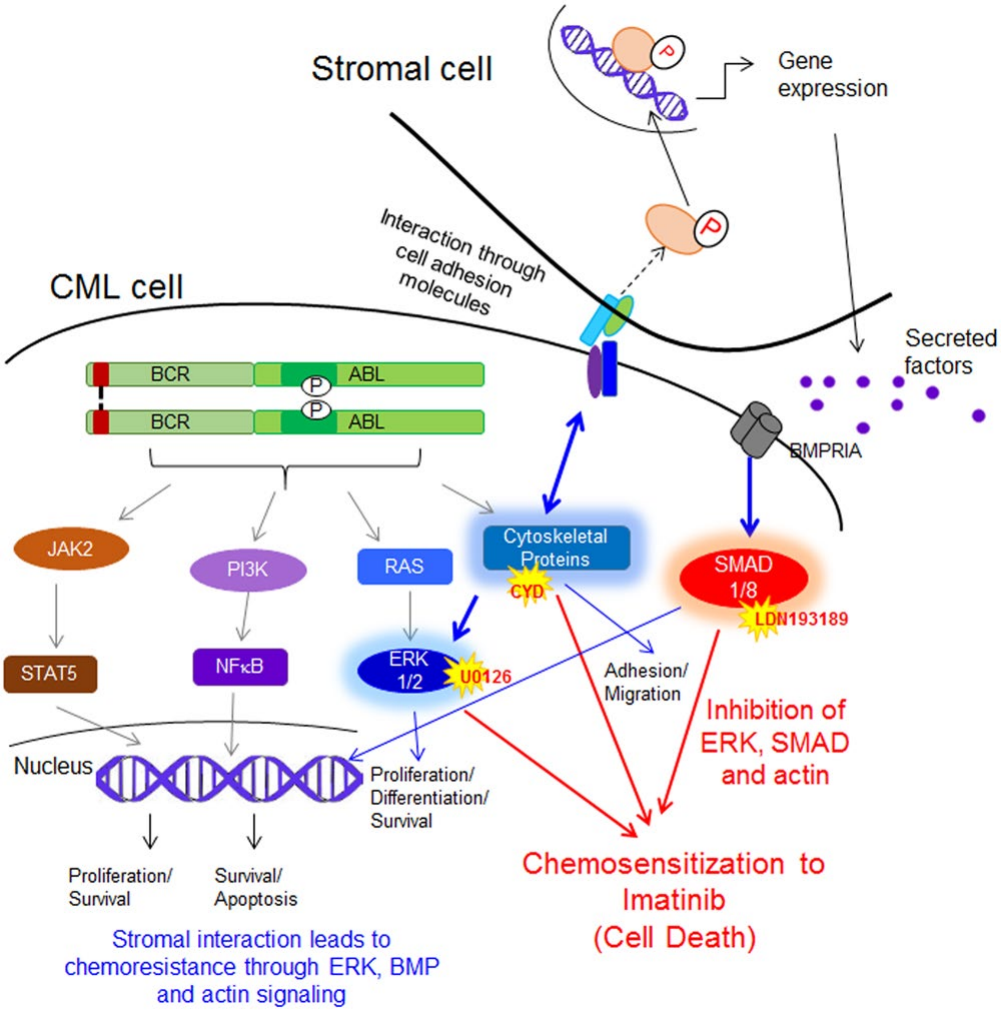
Model representing the development of stroma mediated chemoresistance and signaling molecules that could be targeted for therapy.

Thus, our study shows for the first time, that chemoresistance to IM developed in CML cells through interaction with the microenvironment stromal cells and was dependent on adhesion mediators such as actin cytoskeleton, ERK1/2 MAPK and SMAD signaling (Fig. 9). Inhibition of these pathways abrogated chemoresistance development during IM treatment and sensitized chemoresistant CML cells to IM. Adopting a combinatorial treatment approach will prevent the development of chemoresistance and might eradicate the stroma adherent CML cells and prolong the patient survival.

## Materials and Methods

### Reagents and cell lines

Cytochalasin D, LDN193189, U0126, AMD3100, Bay 11-7082, SB203580, Y27632, 5, 15 DPP and imatinib mesylate were purchased from Sigma Aldrich. Fluorescent dye conjugated antibodies against phosphorylated form of ERK1/2 MAPK, p38 MAPK, NFκB, SMAD1, STAT3 and STAT5 were obtained from BD biosciences. K562 CML cell line was obtained from National Centre for Cell Sciences, Pune. The cell lines were used within 20 passages for the experiments and whenever required, early passage cells were thawed for the experiments. DMEM and RPMI media were purchased from Sigma and fetal bovine serum was from Invitrogen Biosciences.

### Ethical approval

The study was conducted in accordance with the ethical standards described in the Declaration of Helsinki. The study protocol was approved by Ethical committee of Indian Institute of Technology Guwahati and the study was carried out in accordance with the human ethics committee guidelines. Written informed consent was obtained from all the patients involved in the study.

### Primary CML cells

Primary CML cells were isolated from the bone marrow (BM) of patients diagnosed for CML and referred to the Hematology Department of Gauhati Medical College Hospital. The BM samples were transferred to the lab on ice and processed the same day or within 24 hours after collection. First, the cells were subjected to red cell lysis (RBC) using RBC lysis buffer (10 mM Potassium Carbonate, 150 mM Ammonium Chloride, 0.1 mM EDTA, pH 8.0) on ice for 7 minutes. The RBC lysis was terminated by addition of 10% FBS (v/v) and centrifugation. The resulting cell pellet was resuspended in appropriate buffer and mononuclear cells were counted using hemocytometer.

### Patient derived mesenchymal stromal cells

BM cells obtained from CML patients as described above were subjected to RBC lysis and centrifugation. The cell pellet was resuspended in growth media consisting of DMEM low glucose media supplemented with 10% FBS and 1X Penicillin/Streptomycin solution. The cells were plated at a density of 1 × 10^5^ cells/cm^2^ in tissue culture flasks coated with fibronectin (20 ng/cm^2^). Media was changed after 24 hrs and thereafter every 3–4 days until spindle shaped colonies appeared. After the cells reached 70% confluency, they were sub-cultured and used for experiments.

### CML stromal cells co-culture

Stromal cells were grown to confluency in tissue culture treated dishes and leukemic cells were added to the stromal cells at a ratio of 1:10. The cells were co-cultured for the indicated time period. The stromal cells were derived from bone marrow of CML patients and BCR-ABL positive CML cell line K562 and primary patient CML samples in the chronic phase were used for co-culture studies. For separation of adherent leukemic cells from the co-culture, the cells were treated with 0.05% trypsin for 30 seconds while monitoring the cell detachment of leukemic cells under microscope. The detached cells were transferred to a serum containing tube and processed.

### Long-term co-culture

K562 cells were co-cultured with stromal cells for 7 days and suspension cells were discarded. The stroma adherent K562 cells were cultured in presence of 10 μM IM. Fresh IM was added every 72 hrs. Suspension cells were removed at every media change. Co-culture in presence of IM was continued for 10–12 weeks and persistent K562 cells were transferred to fresh stromal layer, stroma free culture or continued on the same stromal layer in the presence of IM.

### Apoptosis analysis

Apoptosis analysis was performed by staining the cells with Annexin-V and PI (Thermo Fisher Scientific) according to the manufacturer’s instructions and analyzed using flow cytometry. Briefly, the cells were collected and washed with ice-cold PBS. The cells were stained with anti -annexin V antibody and propidium iodide (PI) and incubated in dark for 15 mins at room temperature. The stained cells were analyzed with FACS Calibur.

Cell death was further confirmed by staining for active form of caspase-3 and analysis through flow cytometry. Active caspase-3 staining was performed using Active caspase-3 apoptosis kit (BD biosciences) according to the manufacturer’s instructions. After fixation and permeabilization with BD cytofix/cytoperm fixation/permeabilization buffer, the cells were stained with fluorescent conjugated anti-active caspase 3 antibody. The cells were incubated in dark at room temperature for 30 mins, washed and analyzed with FACS Calibur.

### Phospho flow analysis

The phosphorylated levels of signaling proteins were analyzed through phospho flow cytometry by specific staining with antibodies against the phosphorylated forms of the proteins. The cells were fixed with 4% formaldehyde, washed and permeabilized with 100% ice cold methanol. Equal number of cells was stained with fluorescent dye conjugated phopsho specific antibodies (BD Biosciences) at room temperature for 1 hour. The cells were washed with the staining solution and analyzed with flow cytometer.

### Gene expression analysis

Total RNA was extracted using total RNA extraction kit (Invitrogen Biosciences). The RNA was reverse transcribed using high capacity cDNA reverse transcription kit (Invitrogen Biosciences) using oligodT primers. The gene expression at transcript level was assessed by real-time PCR using Power SYBR Green real-time PCR mix (Applied Biosystems) with Applied Biosystems 7500 Real-time PCR system.

### Plasmids and stable cell line preparation

pVSV-G, pCMV-dR8.91, pHR-SIN-RHOAV14, RHOAN19, BMP2 and BMP4 were used to generate cell lines with stable activation and downregulation of RHOA or overexpression of BMP2 and BMP4. 293FT cells (Invitrogen) was used for packaging the lentiviral vector and K562 cells were stably transfected using the lentiviral particles.

### Intracellular IM measurement

The intracellular IM was measured using a fluorimeter as described earlier^34^. Standard curve was prepared using IM stock solution diluted in the lysis buffer and intracellular IM in different cell types was measured.

### Data analysis

Statistical analysis was performed using SPSS software and student t test was used to compare between the treated and untreated groups. Differences in primary patient sample data was analyzed using Mann-Whitney non-parametric variables test. p values < 0.05 were considered statistically significant. Flow cytometric data were analyzed using FlowJo software (FlowJo, LLC). Geometric mean fluorescence intensity (MFI) was calculated to detect the changes in the phospho protein levels. The expression levels were normalized to isotype controls.

## Electronic supplementary material

Supplementary information
