# Supplementary material for: Adhesion to stromal cells mediates imatinib resistance in chronic myeloid leukemia through ERK and BMP signaling pathways
Source: Sci Rep. 2017 Aug 25;7:9535. doi: 10.1038/s41598-017-10373-3 (PMC5572702; doi:10.1038/s41598-017-10373-3)
Supplement: Supplementary file 1 — Supplementary information [file 41598_2017_10373_MOESM1_ESM.pdf]

Adhesion to stromal cells mediates imatinib resistance in chronic myeloid leukemia through ERK and BMP signaling pathways

Atul Kumar, Jina Bhattacharyya, Bithiah Grace Jaganathan

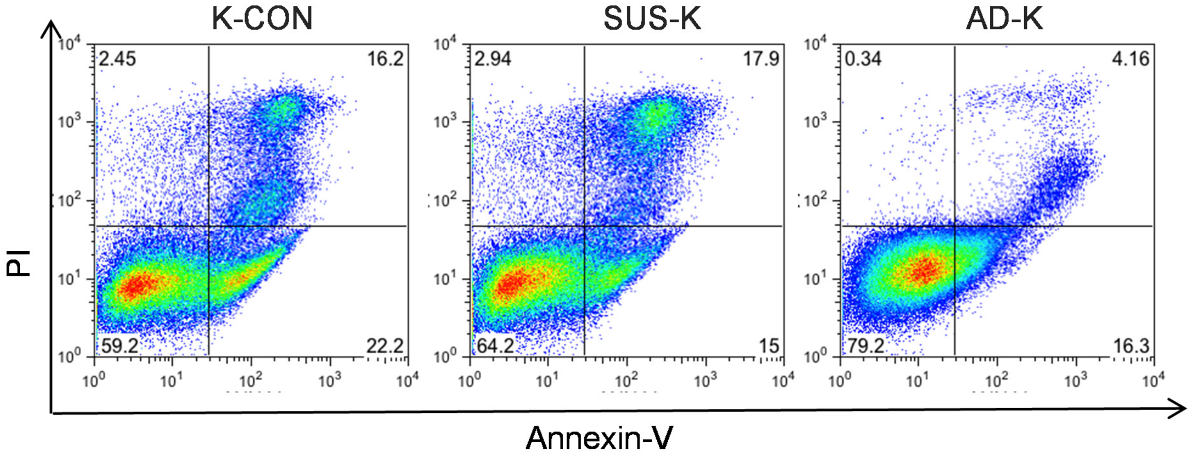

Supplementary Figure 1. Representative flow cytometric plot showing annexin-V/PI analysis of control (K-CON), stroma adherent (AD-K) and suspension (SUS-K) fractions of co-cultured K562 cells treated with IM.

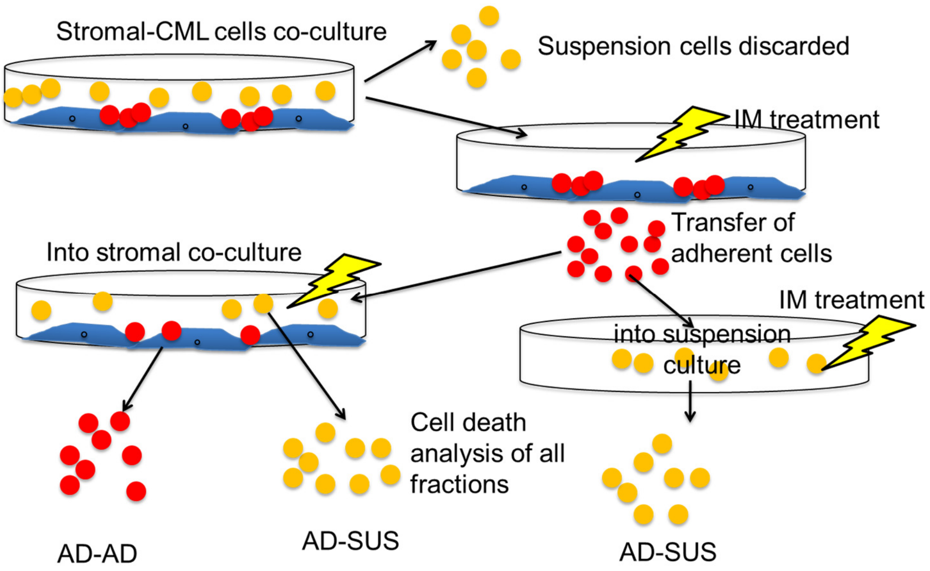

Supplementary Figure 2. Pictorial representation of experimental set-up to analyze the role of cell-adhesion of K562 cells to stroma in chemoprotection against IM treatment for results shown in figure 1f.

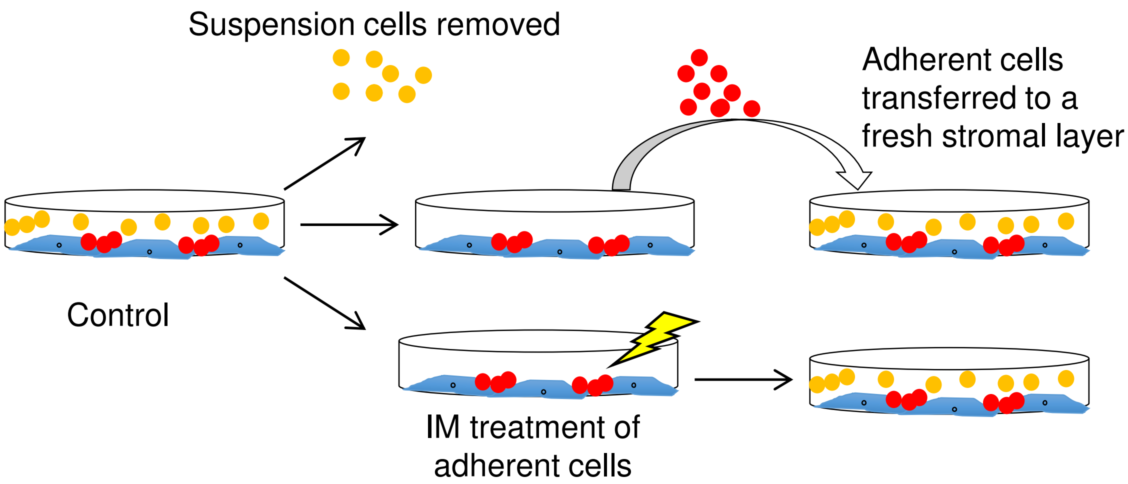

Supplementary Figure 3. Pictorial representation of experimental set-up to analyze the role of cell-adhesion of K562 cells to stroma in chemoprotection against IM treatment for results shown in figure 3.

.
